# Editorial: Metabolomics and Ethnopharmacology in the Development of Herbal and Traditional Medicine

**DOI:** 10.3389/fphar.2022.851023

**Published:** 2022-03-31

**Authors:** Sayeed Ahmad, Chandra Kant Katiyar, Gudrun S. Ulrich-Merzenich, Pulok Kumar Mukherjee

**Affiliations:** ^1^ Bioactive Natural Product Laboratory, Department of Pharmacognosy and Phytochemistry, School of Pharmaceutical Education and Research, Jamia Hamdard University, New Delhi, India; ^2^ Emami Ltd., Kolkata, India; ^3^ Medical Clinic III, AG Synergy Research and Experimental Medicine, University Hospital Bonn, Bonn, Germany; ^4^ School of Natural Product Studies, Jadavpur University, Kolkata, India; ^5^ Institute of Bio-Resources and Sustainable Development (IBSD), Imphal, India

**Keywords:** ethnopharmacology, medicinal plants, AYUSH DRUGS, metabolomics, traditional medicine, herbal medicine

Herbal and traditional medicines are again gaining popularity around the world for the management of a variety of health problems. As the use of herbal/traditional medicines and other botanical products grows and more new products are introduced to the market, new and efficient methods to assess safety and efficacy become a major concern for researchers in drug development. It is important to understand the composition, safety, effectiveness, drug–drug or drug–food interactions, and also the possible side effects of traditionally used herbal medicines. The safety and efficacy data for herbal and traditional medicines are presently insufficient to meet the requirements for their worldwide use.

Traditional medicines are derived from local and empirical knowledge often transmitted orally and have been developed over generations. The traditional healers of Indian AYUSH (Ayurveda, Yoga, Naturopathy, Unani, Siddha, Sowa-Rigpa, and Homeopathy), traditional Chinese medicine (TCM), and other traditional systems of medicine mainly emphasize on holistic approaches with a focus on therapeutic practice and outcomes.

Metabolomics and the metabolomic profiling of herbal medicines and medicinal plants have provided new avenues of research in drug development. Metabolomic approaches allow the simultaneous identification of thousands of metabolites present in medicinal plants or herbal medicines. Furthermore, these support the mechanistic understanding of the concept of multicomponent multitarget effects and polypharmacology as well as the holistic approaches of treatments as per the AYUSH and other traditional systems of medicine. Synergistic effects are often claimed in experimental and clinical studies using multicomponent herbal preparations. Incorporating omics technologies into the assessment of the mode of action of herbal and traditional medicine will help in rationalizing the biological effects by unfolding the possible mechanism of synergistic actions. Such tools will allow us to explore the possibilities of standardization of multi-component extracts and formulations, for dereplication, and prediction of toxicity and safety of herbal preparations. Such a system biology approach should include network pharmacology assessments as well.

Metabolomics is one of the techniques used to identify targeted and untargeted metabolites for scientific validation and the development of herbal and traditional medicine, especially by using high-throughput screening methods. The probability of repetition or working on numerous samples with similar active metabolites is one of the challenges of natural product-based drug development. To overcome these challenges, various “dereplication” methods have been developed. A metabolomic technique combined with a suitable biostatistical method has the ability to facilitate the fast analysis of composite data generated by high-throughput screening.

The scope of this special issue on ethnopharmacology and metabolomics is to focus on the quality, safety, and efficacy of traditional, specially AYUSH, and herbal medicines with more emphasis on metabolomic studies using hyphenated techniques. This issue contains four review articles and 29 research articles by different research groups around the globe on related topics.

Fu et al. studied the active constituents and mechanism of action of the Fuzheng Huayu formula (FZHY), which is a traditional Chinese medicine used for liver fibrosis. The main active components of FZHY are salvianolic acid B, schisantherin A, and amygdalin, which significantly improve the condition of CCl4- and BDL-induced fibrotic liver in rats and mice. In addition to this, the formulation inhibits the activation of hepatic stellate cells by inactivating notch signaling. The authors conclude that the results provide promising scientific evidence for further investigations with the ultimate goal to bring the formula into clinical research as a potential candidate for liver fibrosis.

*Dendrobium officinale* Kimura & Migo (Orchidaceae) polysaccharide is used in Chinese medicine for liver protection and hypoglycemic action. Qu et al. studied the *in vitro* and *in vivo* effects of *D. officinale* polysaccharides in insulin resistance and abnormal lipid metabolism. The results of the study proved that this traditional medicine may serve as a potential therapeutic agent for obesity-related insulin resistance and lipid metabolism.

Kulyal and group studied the variability in secondary metabolites across five cultivars of *Curcuma longa* L. and two cultivars of *C. aromatica* Salisb. The analysis was carried out on rhizomes and essential oils using gas chromatography–mass spectrometry (GC–MS) and liquid chromatography–mass spectrometry (LC–MS) methods. Among many compounds detected by the authors, 28 compounds were common in all seven cultivars and 39 new metabolites were detected from all seven species.

The work of Yan et al. includes the effect of Huanglong granule (HL Granule) in acute asthma and the possible underlying mechanism of action on mice. Changes in lipid composition were identified using UHPLC-Q-exactive Orbitrap MS with a focus on pulmonary lipid homeostasis. In positive and negative ion modes, a total of 304 and 167 lipids, respectively, were identified in lung tissues, with 162 and 109 lipids significantly elevated in the model group. The authors observed that the HL Granules reversed 104 and 73 lipids, respectively, with a statistical difference (false discovery rate <0.05). It was concluded that lipid homeostasis plays an important role in asthma, and HL Granule might be further investigated and developed as an adjuvant therapy for acute asthma.

The effect of *Lysimachia candida* Lindl. on fatty liver disease in rats was investigated by Kamboj et al. The majority of the metabolites found in both control and treatment groups were related to lipid metabolism. Treatment with *L. candida* extract improved the control of lipid metabolism and reversed the metabolic syndrome phenotype in rats.

Zhao et al. investigated the mechanism of a traditional Chinese medicine, Erchen decoction. The composition and function of gut microbiota in obesity and its relation with lipid metabolism disorders were investigated. The underlying mechanism of obesity due to changes in the composition of gut microbiota was also studied by the authors. The Erchen decoction reduced body weight, improved insulin resistance and lipid metabolism, and reduced the concentration of free fatty acids released from white adipose tissue due to excessive lipolysis in rats. This study supports the hypothesis that the basis for treatment of obesity lies in changes in gut microbiota.

According to Liu et al., the traditional Chinese medicine Ramulus mori (Sangzhi) alkaloid (SZ-A) tablets improved the overall metabolic profile in mice, including glucose metabolism with enhanced insulin response, and also improved lipid metabolism, which was collectively linked to the modulation of gut microbiota.

Sharma et al. worked on three different dosage forms of Guduchi stem, *i*.*e*., *Tinospora cardifolia* (Willd.) Miers, by adopting the Ayurvedic pharmaceutical process of Bhavana (levigation). The findings of this study indicated the effectiveness of Svarasa Bhavita Guduchi Churna and Kwatha Bhavita Guduchi Churna in the treatment of diabetes mellitus. This study provides scientific support to Ayurvedic claims that the Bhavana process has pharmaceutico-therapeutic significance in Ayurvedic drug development.

Zahiruddin et al. used response surface methodology to optimize the ratio of aqueous extracts of *Phyllanthus emblica* L., *Piper nigrum* L., *Withania somnifera* (L.) Dunal, and *T. cordifolia* (Willd.) Miers for the development of a combination formulation. The developed polyherbal formulation showed significant immunomodulatory activity on cyclophosphamide-induced immunosuppressed mice. The metabolomic study showed more than 180 metabolites, identified through LC–MS in the optimized combination. Polyherbal combination treatment significantly (*p* < 0.01) enhanced the subsets of immune cells such as NK cells, B cells, CD4 cells, and CD8 cells.

*Berberis aristata* DC. and *Nigella sativa* L. are plants traditionally used for several diseases. Mazhar et al. describe a method of standardization of *B. aristata* and *N. sativa* and investigated their anticancer activity in a 7,12-dimethylbenz [a]anthracene (DMBA)-induced mouse model. Molecular docking was carried out for the marker compounds of both plants with metabolomic studies of essential oils using GC–MS. The study showed that the extracts of *N. sativa* and *B. aristata*, as well as their marker compounds, showed an antitumor activity and had no harmful effects on female mice. Furthermore, they protected against DMBA-induced tumor in a mouse model.

*Sigesbeckia orientalis* L. (syn.: *Siegesbeckia orientalis* L.) (SO) is a remedy in TCM used to reduce the symptoms of joint disorders. It is a toxic herb, but it is hypothesized that by processing, according to TCM theory, its toxicity can be lowered. Jiang and co-authors demonstrated using metabolomics that the raw SO causes pulmonary toxicity and that by processing with rice wine its toxicity is reduced. This supports the classical SO processing theory with scientific evidence.

*Anadenanthera colubrina* (Vell.) Brenan, a plant with antifungal and anti-inflammatory properties, was studied by Maia et al. for its antifungal activity against *Candida albicans*, *C. glabrata*, *C. tropicalis*, and *C. dubliniensis* using the broth microdilution method. Antifungal activity was assessed in terms of antibiofilm effects, proteolytic enzyme activities, viability assays, gene expression, and cytokine expressions. The extract showed a significant antifungal activity against different *Candida* strains with low toxic effects to the host cells.

Salunkhe et al. formulated two formulations of spray-dried alcoholic extracts from three different herbs, namely, *Trigonella foenum-graecum* L, *Momordica charantia* L, and *Cinnamomum verum* J. Presl. The oral bioavailability and pharmacokinetic profile of the formulations were evaluated in terms of their markers diosgenin, charantin, and hydroxychalcone in male Wistar rats. Maximum oral bioavailability was found for charantin, followed by diosgenin and then hydroxychalcone. A significant increase in bioavailability of all the markers was observed after the addition of piperine.

Supercritical fluid extraction is one of the extraction methods with the advantages of short extraction time and high purity of extracts. Mishra et al. described the metabolite profile of supercritical CO_2_ extracts of the Indian variety of *Ophiocordyceps sinensis*, earlier known as *Cordyceps sinensis*, using high-performance thin-layer chromatography and GC–MS, followed by chemometric analysis. The extract was found effective against *Escherichia coli* and *Salmonella typhi* by the generation of reactive oxygen species and can be utilized in mycotherapeutics for multiple bioeffects.

Wang et al. explored the dose–effect/toxicity relationships between the high and low doses of the lower polar fraction of *Bupleuri Radix* (root of *Bupleurum chinense* DC) and its mechanism using liver metabolomics in chronic unpredictable mild stress (CUMS) rats. The median toxicity dose and effective safe dose of *Bupleuri Radix* which caused liver injury at a high dose and psychiatric diseases at a low dose were calculated.

Tene et al. investigated the cardioprotective effect of *Dillenia pentagyna* Roxb. (DP) against doxorubicin (Dox)-induced cardiotoxicity *in vitro* as well as *in vivo.* The finding suggested that the phenol-rich extract/fractions of DP helped in alleviating Dox-induced cardiotoxicity. The LC–quadrupole time-of-flight electrospray ionization MS analysis of bioactive extract/fractions indicated that polyphenols like gallic acid, syringic acid, and sinapic acid could be responsible for the potent cardioprotective effect due to their antioxidant properties.

Erukainure et al. investigated the cardioprotective mechanisms of a traditional medicinal plant of South Africa, *Turbina oblongata* (E. Mey. ex Choisy) A. Meeuse. The results indicated that such plant has the potential to mitigate lipotoxicity and control dysregulated cardio-metabolic activities due to its antioxidant potential and suppressive effects on angiotensin-converting enzyme, lipase, and acetyl cholinesterase enzymes.

Mahuang decoction (MHD) is a well-known traditional Chinese medicine; its protective effect against lipopolysaccharide and D-galactosamine (LPS/D-GalN)-induced acute liver failure (ALF) in a mouse model was reported by Liao and his co-workers. The MHD showed a protective effect by regulating the tricarboxylic acid cycle and amino acid metabolism. Ultra-performance liquid chromatography (UPLC)–MS was undertaken for metabolomic studies, and it revealed that, in serum samples, 36 metabolites were identified as contributing to LPS/D-GalN-induced ALF, whereas 27 among them were ameliorated on the administration of MHD.

Erukainure et al. worked on the tetrahydrocannabinol-rich extract of *Cannabis sativa* L. in the context of neurodegenerative disorders. The extract was found to improve glucose intake and to suppress oxidative stress and cholinergic dysfunction as well as to modulate purinergic and gluconeogenic activity in the brain tissues of rats. The *in silico* analysis revealed that the constituents of the extract can pass through the blood–brain barrier, whereas the GC–MS analysis confirmed the presence of tetrahydrocannabinol in the plant extract.

Safoof-e-Pathar phori (SPP) is a poly-herbomineral formulation, which has been used traditionally for urolithiasis as per the Unani Pharmacopoeia of India. Ahmad et al. investigated the traditional claim pre-clinically. The study involved the oral administration of SPP at low and high doses. They significantly (*p* < 0.001) reduced urinary calcium, serum creatinine, blood urea, and lipid peroxidation in urolithiatic Wistar rats. The long-term oral toxicity study showed that SPP was safe in Wistar rats for up to 3 months. The study provides scientific evidence in support of traditional claims for SPP as an anti-urolithiatic formulation.

Luo et al. used high-throughput metabolomic analysis to identify the biomarkers and pathways that would reveal the therapeutic action and mechanism of andrographolide against lung cancer using UPLC–time-of-flight MS. The findings suggest that 11 metabolism pathways are regulated by andrographolides in cancer. The network pharmacology revealed the involvement of 570 proteins. Amino acid metabolism and arachidonic acid metabolism pathways are the potential target pathways for andrographolide in this model of lung cancer.

Kumar et al. investigated the neuroprotective effect of piperine on streptozotocin-induced hyperglycemia and also observed the gene expression in diabetic rats. Piperine leads not only to a significant improvement in memory but also to a significant reduction in the expression of specific Alzheimer’s disease-related genes, like BACE1, PSEN1, APAF1, CASPASE3, and CATALASE.

*Radix Bupleuri* (RB) and *Radix paeoniae* Alba (RPA) are components of a well-known herb combination used clinically to treat depression. Chen et al. developed a novel and efficient technique for analyzing the impact of the combination of RB and RPA in *in vivo* behaviors by combining multi-component pharmacokinetics with metabolomics. The finding of the study suggested that the combination can increase the bioavailability of 6 components in RPA and 5 in RB and also boost neuroprotective and anti-inflammatory effects.

Tiwari et al. used chromatographically standardized extracts of *Clerodendrum serratum* (L.) Moon (Verbenaceae) for assessing its anti-inflammatory and anti-arthritic activities. Scientific evidence for the ethnomedicinal use of the plant in arthritis is thereby provided.

Nandanwadkar et al. used the inductive coupled plasma-optical emission spectroscopy technique to carry out multi-elemental assessments of phyto-pigments. They also used a chromatographic technique to evaluate their biotherapeutic potential. The screening for heavy metals and micro- and macro-minerals was undertaken using routine quality control and the safety profile of food additives and contaminants.

Lin et al. focused on two traditional Chinese medicine [*Acanthopanax senticosus* (Rupr. and Maxim.) Harms and *Gastrodia elata* Blume] for the treatment of stroke and cerebrovascular diseases. Using transcriptomic and metabolomic studies together, the authors hypothesized that both drugs can be used for the treatment of cerebrovascular diseases. Six metabolites and six genes were found to be significantly altered. The therapeutic effect of the extracts in cerebrovascular diseases was found to be related to the regulation of the phenylalanine and pyrimidine metabolic pathways.

*Pseudevernia furfuracea* (L.) is an epiphytic lichen used in Indian spice mixtures, curries, and food preparations as a preservative. Goel et al. attempted to find the optimal extraction method for polyphenol- and flavonoid-enriched extracts of *P. furfuracea*. Scanning electron microscopy and high-performance liquid chromatography were used to analyze and compare the effect of pre-processing conditions on the extraction method. Ultra-high-performance liquid chromatography–diode array detector MS was used to study the metabolomic profile of the lichen extracts. After mixing and grinding the raw material by using Soxhlet, it was found that 70% methanol extract was the most effective for extracting a combination of polyphenolic and flavonoidal-rich metabolites. The work also suggested *P. furfuracea* as a potent antioxidant.

Sing et al. suggested a system for grading *Andrographis paniculata* (Burm. F) raw ground samples using near-infrared reflectance (NIR) spectroscopy and support vector machine (SVM) classifier based on the content of the marker, andrographolide. The estimation accuracy based on extracts was marginally higher than that based on powder leaf samples. However, it had no effect on the samples’ grading pattern. The finding of the study suggested that combining the NIR-based estimate of powdered leaf samples with an SVM classifier can be a low-cost solution for grading the samples rapidly.

Mou et al. summarized the structural types, pharmacological activities, and mechanisms of *Dendrobium* alkaloids as well as the suggested biogenetic pathways of dendrobine, which is an important sesquiterpene alkaloid.

Tawfeek et al. provided a comprehensive overview of the phytochemistry, traditional use, and pharmacology of plant extracts and constituents from the genus *Salix* (willows). They also demonstrated its ability to reduce inflammatory pathways and hypothesized that they could be useful in cancer prevention and therapy as well as other chronic diseases.

Bhatt et al. explored the Indian system of medicines for possible male and female contraceptives. The review concluded that the Indian system of medicine offers highly promising opportunities with potential analytical, biological, technological, and clinical advances collectively integrated with therapeutic rationale based on Ayurvedic principles.

Nille et al. provided information on a traditional plant, Avartaki [*Senna auriculata* (L.) Roxb.], and its wide usefulness in the Ayurveda and Siddha systems of medicine for the treatment of numerous diseases. The ethnomedicinal, phytochemical, pharmacological, and toxicological features of the plant were discussed in this review article, with more focus on therapeutic significance in diabetes.

Overall, this special issue provides an insight into the role of metabolomics and ethnopharmacology in the development of herbal and traditional medicine. It provides the readers with a plethora of generally up-to-date information to support their understanding of these rapidly evolving areas of metabolomics. There is still an enormous scope to work on the metabolomics and network pharmacology of traditional medicine for a better understanding of the molecular mechanism of pharmacological actions by identifying metabolites through dereplication and to provide data for scientific validation of traditional claims.

